# Clinical Features and Prognostic Factors in Patients With Uterine Leiomyosarcoma: A Single-Center Experience

**DOI:** 10.7759/cureus.43681

**Published:** 2023-08-18

**Authors:** Merih Yalciner, Hatice Bölek, Elif Berna Köksoy

**Affiliations:** 1 Medical Oncology, Ankara University School of Medicine, Ankara, TUR

**Keywords:** treatment and prognosis, uterine malignancy, uterine neoplasm, uterine leiomyosarcoma, uterine sarcomas

## Abstract

Background

Uterine leiomyosarcomas (LMS) are associated with more recurrence and higher mortality compared to other uterine cancers. Considering the limited number of case series in the literature, the limited effectiveness of standard treatment methods, and the inadequacy of molecular biomarkers, we planned to investigate the effects of treatment methods and survival outcomes in these patients.

Methodology

The study was designed retrospectively, and the records of patients who were followed up and treated at Ankara University Faculty of Medicine, Medical Oncology Clinic, between January 1, 2011, and December 31, 2021, were reviewed. Patients over 18 years of age with a pathological diagnosis of uterine LMS were included. Demographic, clinical, and pathological data were recorded using the hospital database. The International Federation of Gynecology and Obstetrics (FIGO) staging was reassessed for each patient in accordance with the AJCC Cancer Staging Manual, Eighth Edition (2017). Tumor size, location, and grade were also evaluated. Types of treatments, protocols, and adverse effects were recorded. Relapsed patients, relapse localization, and treatments given at relapse were recorded and compared.

Results

Twenty-eight patients were included. The mean age of the patients was 53.7 years. The median follow-up time was 39.3 months. The localization of LMS could be detected in 22 (78.57%) patients, among them 20 (90.9%) patients had intramural, 1 (4.5%) had submucosal, and 1 (4.5%) had subserosal LMS. All patients (26, 92.8%) underwent primary surgery, except for 2 (7.14%) patients who were metastatic at the time of diagnosis. Adjuvant treatment suggestion was made for 7 (25%) patients with a high risk of recurrence in the multidisciplinary tumor council. Partial response was observed in 1 (3.5%) of the 2 (7.1%) metastatic patients, and stable disease was observed in the other. Recurrence was detected in 22 (84.6%) patients . Fifteen (53.6%) patients died during the follow-up period. Survival was better in premenopausal patients (99.2 versus 51.6 months, *P *= 0.056). No significant difference was found when the survival of patients who received and did not receive adjuvant treatment were compared. In relapsed patients, there was no significant difference in survival between patients who underwent and did not undergo surgical treatment.

Conclusions

Uterine LMS is a rare and aggressive malignancy with limited diagnostic methods, frequent recurrences, high mortality, and limited use of nonsurgical treatments. The positive effect of adjuvant treatment on survival has not been demonstrated. Further studies are needed to investigate the effect of hormone receptor status on prognosis and new biomarkers.

## Introduction

Leiomyosarcomas (LMSs), a rare malignancy of the uterus, are linked to greater recurrence rates and higher mortality when compared to endometrial cancers. Uterine sarcomas account for approximately 3% to 7% of uterine cancers, and LMS accounts for only 1% to 2% of uterine malignancies [1]. While a majority (60%) of cases are diagnosed at an early stage, the five-year survival rate ranges from 25% to 76%, and for patients with metastasis at the time of diagnosis, survival drops to approximately 10% to 15% [2]. Apart from the aggressive behavior of the tumor, the nonspecific symptoms and the absence of a diagnostic laboratory or imaging test also complicate the management of the disease, and some of the patients are operated with a prediagnosis of benign etiology, such as uterine myoma [3,4].

Surgery is the preferred treatment for early-stage uterine LMS. The efficacy of adjuvant therapy has not been demonstrated and is not currently recommended in standard therapy. However, a recently published retrospective study reported better disease-free survival in patients who were given adjuvant therapy, although this was not statistically significant [5].

Given the rarity of the disease, the limited number of case series in the literature, the limited effectiveness of standard treatment methods, diagnostic difficulties, and the inadequacy of molecular biomarkers, it can be asserted that further research is necessary to delve into the characteristics of uterine LMS patients and the progression of the disease. In this retrospective study, we planned to investigate the effects of treatment methods on survival, as well as the clinical and demographic characteristics of patients with uterine LMS who were followed up and treated in our center.

## Materials and methods

The study was designed retrospectively, and the medical records of patients with a diagnosis of uterine LMS who were followed up and treated at Ankara University Faculty of Medicine, Medical Oncology Clinic, between January 1, 2011, and December 31, 2021, were reviewed. Patients over 18 years of age with a pathological diagnosis of uterine LMS and whose data could be accessed were included in the study. Demographic data, clinical and pathological data, and treatment methods were recorded using the hospital database. The International Federation of Gynecology and Obstetrics (FIGO) staging was reassessed for each patient by the final staging recommendations. Tumor size, location, and grade were also evaluated. Surgical treatments, type of chemotherapy given, protocols, and adverse effects were recorded. Relapsed patients, relapse localization, and treatments given at relapse were recorded and compared.

Statistical analyses were performed using IBM SPSS Version 26 (IBM Corp., Armonk, NY, USA). The conformity of the variables to the normal distribution was examined by visual (histogram and probability graphs) and analytical methods (Kolmogorov-Smirnov and Shapiro-Wilk tests). Parameters that did not show normal distribution were compared using the Mann-Whitney U-test. The effects of the compared parameters on survival were examined using the log-rank test. Survival rates were calculated using Kaplan-Meier survival analysis. Cases where the type 1 error level was below 5% were considered statistically significant.

## Results

Twenty-eight patients were included in the study. The mean age of the patients was 53.7 years. Twelve (42.9%) patients were premenopausal and 16 (57.1%) were postmenopausal. The median tumor size was recorded as 6.25 cm. The localization of LMS could be detected in 22 (78.57%) patients, among them 20 (90.9%) patients had intramural, 1 (4.5%) had submucosal, and 1 (4.5%) had subserosal LMS. Ten (35.7%) tumors were of high grade. Two (3.6%) patients had metastases at the time of diagnosis. The distribution of patients according to stages and tumor markers is summarized in Table 1.

**Table 1:** Characteristics of patients diagnosed with uterine leiomyosarcoma. SD, standard deviation

Variables	
Age (mean ± SD) (years)	53.7 ± 11.7
Tumor size (cm), median (min-max)	6.25 (1.8-18)
Serum LDH, median (min-max)	196.5 (10-1,131)
Serum CA-125, median (min-max)	24.8 (7.4-384)
Ki-67, median (min-max)	40 (20-80)
Menopause status, *n* (%)	
Premenopausal	12 (42.9)
Postmenopausal	16 (57.1)
Location, *n* (%)	
Intramural	20 (71.4)
Submucosal	1 (3.6)
Subserosal	1 (3.6)
Unknown	6 (21.4 )
Tumor grade, *n* (%)	
1	0 (0)
2	1 (3.6)
3	10 (35.7)
Unknown	17 (60.7)
Presence of metastases at diagnosis	2 (7.1)
Stage, *n* (%)	
1A	8 (28.6)
1B	15 (53.6)
4B	2 (7.1)
Unknown	3 (10.7 )

All patients (26, 92.8%) underwent primary surgery, except for 2 (7.14%) patients who were metastatic at the time of diagnosis. The types of surgery are summarized in Table 2. When the surgical notes and pathology reports were examined, it was observed that complete resection was achieved in all patients who underwent primary surgery. Adjuvant treatment suggestion was made for 7 (25%) patients with a high risk of recurrence (high tumor burden, positive lymph nodes, proximity to surgical margin, and advanced FIGO stage) in the multidisciplinary tumor council. Subsequently, these seven patients underwent adjuvant therapy using the ifosfamide + mesna + adriamycin (IMA) protocol. The applied treatment protocols and adverse effects are summarized in Table 2. 

**Table 2:** Treatment modalities and adverse effects in patients with uterine LMS. LMS, leiomyosarcoma; USO, unilateral salpingo-oophorectomy; BSO, bilateral salpingo-oophorectomy; PLND, pelvic lymph node dissection; IMA, ifosfamide + mesna + adriamycin

Variables	*n* (%)
Number of patients underwent primary surgery	26 (92.8)
Surgery type	
USO	1 (3.8)
USO + hysterectomy	1 (3.8)
BSO + hysterectomy	12 (46.1)
BSO + hysterectomy + PLND	11 (42.3)
Myomectomy	1 (3.8)
Complete resection	26 (100)
Patients diagnosed after surgery	10 (35.7)
Systemic treatment	
Adjuvant	7 (87.5)
Palliative	1 (12.5)
Systemic treatment protocols	
IMA	7 (87.5)
Gemcitabine + Docetaxel	1 (12.5)
Adverse effects	
Neutropenia	
Grade 1	1 (12.5)
Grade 2	1 (12.5)
Anemia	
Grade 1	1 (12.5)
Grade 4	1 (12.5)
Thrombocytopenia	
Grade 4	1 (12.5)
Nausea and vomiting	
Grade 1	2 (25)
Grade 2	1 (12.5)
Adverse events leading to treatment discontinuation	1 (12.5)

Partial response was observed in one of two metastatic patients, and stable disease was observed in the other. Recurrence was detected in 22 (84.6%) patients after surgery. Surgical and systemic treatments applied in relapsed patients are summarized in Table 3. It was recorded that 15 (53.6%) patients died during the follow-up period.

**Table 3:** Treatment modalities after relapse. IMA, ifosfamide + mesna + adriamycin

Variables	*n* (%)
Number of patients who relapsed after surgery and/or adjuvant therapy	22 (84.6)
Surgery in relapsed patients	8 (36.4)
Complete resection after surgery in relapsed patients	6 (75)
Systemic treatment in relapsed patients	19 (86.4)
Treatment protocols in relapsed patients	
First-line IMA	13 (68.4)
Second-line IMA	3 (15.7)
Second-line Gemcitabine + Docetaxel	3 (15.7)
Death	15 (53.6)

**Figure 1:**
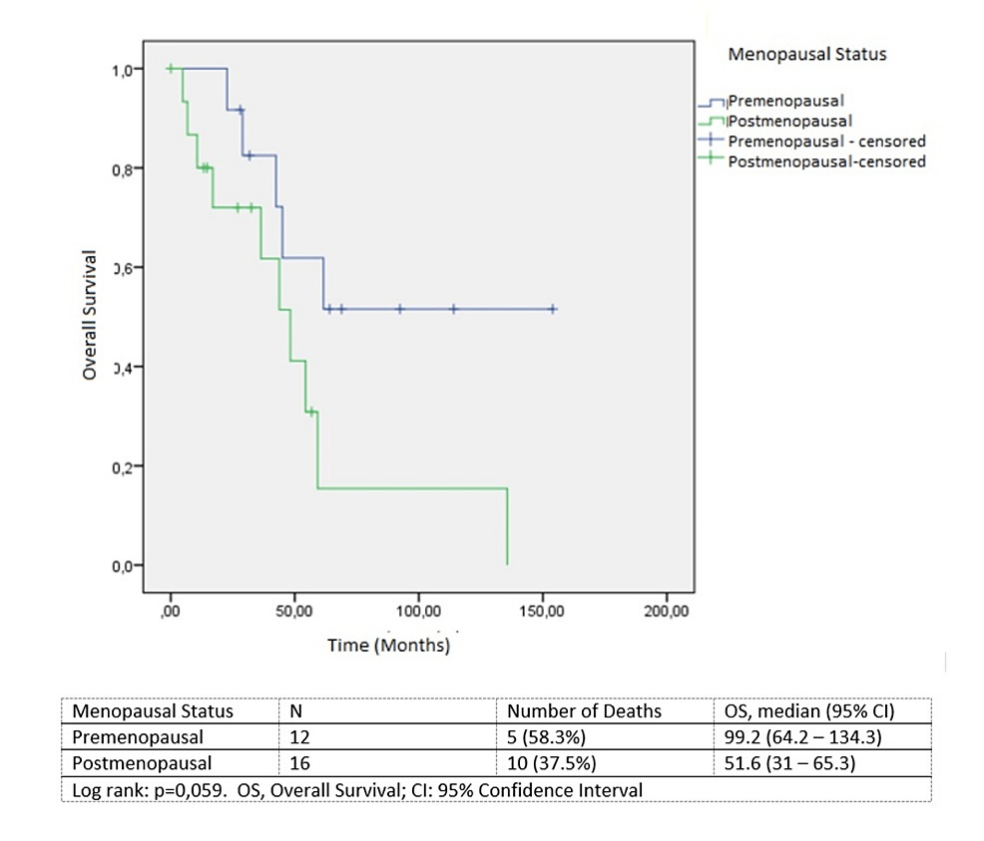
Overall survival by menopausal status.

Survival by menopausal status is summarized in Figure 1. Survival was better in premenopausal patients (99.2 versus 51.6 months, *P *= 0.056).

The relationship between Ki-67, serum lactate dehydrogenase (LDH), and CA-125 levels and mortality is summarized in Table 4. No statistically significant relationship was observed between these parameters and mortality.

**Table 4:** Relationship between tumor markers and laboratory characteristics and mortality. ^*^Mann-Whitney U-test. LDH, serum lactate dehydrogenase level

Mortality			
Variables	Present (*n* = 15, 53.6%)	Absent (*n* = 13, 46.4%)	*P*-value
Ki-67 (median ± min-max)	40 (30-50)	60 (30-80)	0.19*
LDH (median ± min-max)	218 (139-485)	159 (10-1,131)	0.17*
CA-125 (median ± min-max)	49 (8-120)	19.6 (7.4-91)	0.56*

Survival time was statistically significantly shorter in advanced disease (*P *= 0.001). Post hoc analyses showed that the difference was due to patients with Stage 4B.

**Figure 2:**
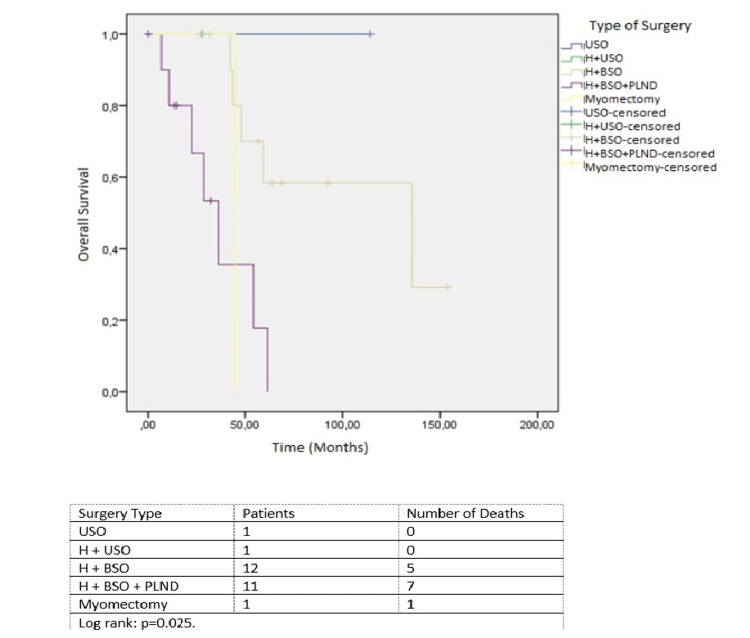
Relationship between surgery type and overall survival. USO, unilateral salpingo-oopherectomy; BSO, bilateral salpingo-oopherectomy; PLND, pelvic lymph node dissection; H, hysterectomy

The relationship between surgery type and overall survival is shown in Figure 2.

The number of patients diagnosed with biopsy before surgery was 18 (64.2%), and the number of patients diagnosed after surgery was 10 (35.8%). The median survival of the patients is summarized in Table 5. There was no statistically significant relationship between the diagnosis before or after surgery and survival.

**Table 5:** Survival data of patients with and without preoperative biopsy diagnosis. Log-rank, *P *= 0.7. OS, overall survival; CI, confidence interval

Presurgical diagnosis	Patients	Number of deaths, *n* (%)	OS, median (95% CI)
Yes	18	9 (50)	61.5 (41.9-81.1)
No	10	6 (40)	44.9 (41.6-48.3)

No statistically significant difference was found when the survival of patients who received and did not receive adjuvant systemic treatment was compared.

There was no statistically significant relationship between the site of recurrence and survival. The findings about survival by location of relapse are summarized in Table 6.

**Table 6:** Survival by location of relapse. Log-rank, *P *= 0.9. OS, overall survival; CI, confidence interval

Location of relapse	Patients	Number of deaths	OS, median (95% CI)
Local	5	3	44.9 (8.3-61.3)
Intraabdominal	5	3	43.7 (0-113.4)
Distant metastasis	10	5	54.3 (9.8-98.7)
Local + distant metastasis	2	2	48.1 (41.7-67.9)

In relapsed patients, there was no statistically significant difference in overall survival between patients who underwent and did not undergo surgical treatment (59.1, 95% CI 23.3-95 vs 48.1, 95% CI 29.9-66.5; *P *= 0.77). As the number of patients who did not receive systemic therapy at relapse was only two, patients who received and did not receive systemic therapy could not be compared. There was no significant difference in survival time between systemic treatment regimens (*P *= 0.53).

## Discussion

The fact that uterine LMS is a very rare disease makes it difficult to conduct a single-center study. However, the literature contains a limited number of case series, and the controversy persists regarding optimal systemic treatment, treatment timing, and the benefits of adjuvant therapy. Additionally, scant information exists concerning the prediction of the disease prognosis. In addition to being rare, it is known that the mortality of the disease is also high. In a study that included 62 uterine sarcoma patients, it was shown that uterine sarcomas have a poor prognosis and uterine LMS was a poor prognostic histological variant among them [6].

The median age of the patients in our study was 53 years. Twelve (42.9%) patients were premenopausal and 16 (57.1%) were postmenopausal. The mean age and the frequent occurrence of the disease in the perimenopausal period are consistent with other series in the literature [3,4,7]. Furthermore, we observed that the survival of premenopausal patients was better. Although this finding is consistent with previous studies [3,4], its reason has not been fully explained. One possible explanation could be that postmenopausal patients tend to have more comorbidities or lower performance status. However, the fact that the cause of mortality in our series was disease progression, rather than additional comorbidities or chemotherapy side effects, implies that this explanation is not fully satisfactory.

Although some studies have reported that serum LDH and CA-125 levels may be high [2], it is known that no tumor marker can be used routinely in the diagnosis and follow-up of uterine LMS. In our study, it was seen that these markers and Ki-67 levels were not associated with survival. Consistent with the findings of our study, it is understood that the most important predictor of prognosis is the stage of the disease. In addition, potential biomarkers for differential diagnosis of the disease are continually being investigated, although there is not yet a noninvasive technique to diagnose uterine LMS [8,9]. It is also known that the differential diagnosis of leiomyoma with imaging can be difficult [10]. These findings indicate that new biomarkers should be investigated in the follow-up and treatment of uterine LMS.

When the patients were evaluated according to the types of surgery, it was observed that the group with the lowest median survival was the patients who underwent hysterectomy combined with bilateral salpingo-oophorectomy and pelvic lymph node dissection. This finding can be explained by the fact that this surgery was performed in patients with more advanced disease stages. A recently published meta-analysis showed limited benefit of lymphadenectomy in early-stage uterine LMS [11]. On the other hand, it was seen that there was only one patient who underwent myomectomy in primary surgery, and this patient died. Although there was no significant difference in survival between patients diagnosed before or after surgery in our study, this finding may be due to the low number of patients. It is known that the differential diagnosis of benign tumors and LMS can be difficult with imaging methods [12]. These findings suggest that further studies are needed to develop imaging modalities for the diagnosis of LMS.

As mentioned earlier, adjuvant therapy is not currently recommended as a standard for LMS and the place of adjuvant therapy is still controversial. In our study, there was no difference in survival between patients who received and did not receive adjuvant therapy. In a recent retrospective study involving 86 patients [5], it was shown to have a positive effect on disease-free survival. In other studies [13,14], adjuvant therapy was reported to not affect disease-free and overall survival. However, in a recently published meta-analysis, it was reported that a history of neoadjuvant chemotherapy and local radiotherapy in earlier stages of the disease have an improvement in the outcome of recurrent uterine LMS receiving current therapy [15].

In our study, no difference in survival was observed between the sites of relapse and the systemic treatment regimens given at relapse. These findings also support that the stage of the disease and its operability at the time of diagnosis are the most important parameters in terms of prognosis.

In addition, it has been reported in the literature that obesity, menopausal use of estrogen plus progestin, oral contraceptives, and tamoxifen use are associated with uterine sarcomas [7,8]. Further studies are needed to investigate the effect of hormone receptor status on prognosis and new biomarkers.

Our study's limitations comprise the small sample size and its retrospective design.

## Conclusions

Uterine LMS is a rare and aggressive malignancy with limited diagnostic methods, frequent recurrences, high mortality, and limited use of nonsurgical treatments. In recent years, significant improvements in survival have not been achieved. The primary treatment of the disease is surgery, and the positive effect of adjuvant treatment on survival has not been demonstrated. Further studies are needed to investigate the effect of hormone receptor status on prognosis and new biomarkers.
